# Incidence, clinical characteristics, and survival outcomes of ovarian strumal diseases: a retrospective cohort study

**DOI:** 10.1186/s12905-023-02624-5

**Published:** 2023-09-19

**Authors:** Sijian Li, Ruping Hong, Min Yin, Tianyu Zhang, Xinyue Zhang, Jiaxin Yang

**Affiliations:** 1 National Clinical Research Center for Obstetric and Gynecologic Diseases, Department of Obstetrics and Gynecology, Peking Union Medical College Hospital, Chinese Academy of Medical Sciences, Peking Union Medical College, Beijing, People’s Republic of China; 2 Department of Pathology, Peking Union Medical College Hospital, Chinese Academy of Medical Sciences, Peking Union Medical College, Beijing, People’s Republic of China; 3 Department of Gynecology, Cheeloo College of Medicine, Qilu Hospital (Qingdao), Shandong University, Qingdao, People’s Republic of China

**Keywords:** Struma ovarii, Ovarian strumal carcinoid, Malignant struma ovarii, Clinical characteristics, Survival outcomes

## Abstract

**Background:**

Struma ovarii (SO) is a rare tumor and may transform into ovarian strumal carcinoid (OSC) and/or malignant struma ovarii (MSO), but the incidence, clinical characteristics, and survival outcomes have not been well defined.

**Methods:**

We conducted a retrospective study of patients with ovarian strumal diseases treated in the our hospital between 1980 and 2022. Subgroup analyses of SO, OSC, and MSO were subsequently performed.

**Results:**

A total of 275 cases (2.14%) were identified in a cohort of 12,864 patients with ovarian teratomas, where SO, OSC, and MSO accounted for 83.3%, 12.0%, and 4.7% of cases, respectively. There were no significant differences in age, tumor sizes, elevated tumor markers, and ascites among the three subgroups. At initial treatment, all patients with SO or OSC had FIGO stage I disease except one SO patient presenting metastatic disease, ten patients had MSO confined to the ovary, whereas other three patients had metastatic diseases. Two patients with SO respectively relapsed at peritoneum and anterior mesorectum, while none of the OSC patients presented tumor recurrence or death despite different surgical procedures employed. The 5-year recurrence-free survival rate was 88.9%, and only one death occurred at 9.5 years after diagnosis in patients with MSO. Radioiodine therapy showed satisfactory therapeutic efficacy, but these patients showed poor responses to the chemotherapy.

**Conclusion:**

2.14% of ovarian teratoma could be classified as SO, of which 12.0% and 4.7% of SO may transform into OSC and MSO, repsectively. The survival outcomes were excellent even after SO transformed into OSC or MSO.

**Synopsis:**

SO occupied 2.14% of ovarian teratoma, where 12.0% and 4.7% of SO may transform into OSC and MSO, respectively, and had excellent survival outcomes.

**Supplementary Information:**

The online version contains supplementary material available at 10.1186/s12905-023-02624-5.

## Introduction

Struma ovarii (SO) is a monodermal teratoma, characterized by the presence of more than 50% of thyroid tissue, which accounts for about 5% of ovarian teratomas and less than 1% of all ovarian tumors [1, 2]. Its clinical manifestations include asymptomatic ovarian mass, abdominal discomfort, thyrotoxicosis, ascites, elevated CA125, or even metastasis, presenting unpredictable biological behaviors with overlapping benign and malignant features [3–5]. Malignant transformation of SO to an ovarian strumal carcinoid (OSC) or malignant struma ovarii (MSO) is also reported in rare circumstances [6, 7].

However, most studies concerning SO, OSC, or MSO were either case reports or small-scale cohort studies [8–10], and the exact incidence of SO and the probability of malignant transformation to OSC or MSO remains unclear. Moreover, the clinical characteristics and prognoses of OSC and MSO have not been well-defined. Besides, accumulated evidence from case reports may bias a comprehensive understanding of this disease due to the significant heterogenicity. Recently, Wei et al. [11] investigated the pathological characteristics in 96 cases, and Savelli et al. [4] evaluated ultrasonic features in 31 SO cases, which were the two largest cohorts to date but without reporting any surgical options. Several cohort studies of OSC were either conducted decades earlier or restricted to pathologic research, or lacking long-term follow-up [12–14]. Moreover, none of these studies compared the clinical characteristics among patients with SO, OSC, and MSO in large cohorts.

To evaluate the incidence, clinical characteristics, and survival outcomes of patients with ovarian strumal diseases, we conducted a single-center retrospective study. Subgroup analyses of patients with SO, OSC, or MSO were also performed.

## Materials and methods

The Ethics Committee of the Peking Union Medical College Hospital (PUMCH) approved this study. We defined ovarian strumal diseases as a group of ovarian tumors that contain SO-related components, including SO, OSC, and MSO in this study. Patients with ovarian strumal diseases treated in the PUMCH from January 1st 1980 to December 1st 2022 were included in this study. We screened all patients with the ICD-10 code for SO (ICD-10 code: D39.1 M9090/0), OSC (ICD-10 code: D39.1 M9091/1), and MSO (ICD-10 code: D39.1 M9090/3) in their medical records. After reviewing the patient’s medical records, data including the demographics, clinical and pathological features, treatments, and survival outcomes, were extracted from eligible cases. Twelve patients who lacked any of such items were excluded after the screening (Figure S1 summarized the inclusion process). Pathological review was conducted to confirm the diagnosis in OSC or MSO patients diagnosed before 2000. Surgical options were classified as ovarian cystectomy, unilateral salpingo-oophorectomy (USO), bilateral salipingo-oophorectomy (BSO), hysterectomy with BSO (H/BSO), and staging/cytoreductive surgery. We defined staging/cytoreductive surgery as H/BSO plus omentectomy with or without lymphadenectomy/appendectomy/metastasectomy. Clinical features, including age, tumor size, elevated tumor markers (yes or no), preoperative diagnosis (benign, suspected malignant/undetermined, or obvious malignant), and ascites (yes or no), were compared among the three subgroups. Recurrence-free survival (RFS) was defined as the date from initial treatment intervention to confirmed tumor relapse. Overall survival (OS) was defined as the time from the date of initial diagnosis to death associated with any cause or final follow-up. Disease-specific survival (DSS) was defined as the time from the date of the initial diagnosis to death related to the tumor or final follow-up.

### Statistical analysis

Continuous variables were described as means ± standard deviation (range) or as medians and interquartile ranges (IQRs), according to their distributions. Discrete variables were expressed as counts (percentages). One-way analysis of variance (ANOVA) was used to identify differences between the three subgroups. Categorical variables were compared either by the chi-squared testor or Fisher’s exact test. The Kaplan–Meier method was used to establish survival curves. A two-tailed P value < 0.05 was considered statistically significant. Statistical analyses were conducted using SPSS (version 21.0; SPSS Inc., Chicago, IL, USA) or GraphPad Prism (version 8.0; GraphPad Software Inc., La Jolla, CA) software.

## Results

### Clinical characteristics of the patients with ovarian strumal diseases

A total of 275 (2.14%) patients with ovarian strumal diseases were identified in 12,864 patients diagnosed with ovarian teratomas. The median age of the patients was 43 years (range: 17–92). Two hundred and twenty-nine patients (83.3%) were diagnosed with SO, being the most predominant pathologic subtype. Notably, OSC was less common, occupying 12.0% (33 cases) and MSO was rarely seen in only 4.7% (13 cases) in this cohort (Table 1).

**Table 1:** Clinical characteristics in overall cohort and the three subgroups

	SO (N = 229, 83.3%)	OSC (N = 33, 12.0%)	MSO (N = 13, 4.7%)	Overall (N = 275)
Age (y)	44.1 ± 14.7/43.0 (17-92)	44.2 ± 12.0/44.0 (23-72)	42.2 ± 13.8/42.0 (22-78)	44.1 ± 14.3/43 (17-92)
Follow-up time (y)	7.3/5.6 (0.40-32.20)	5.0/3.1 (0.25-25.90)	8.8/6.2 (0.80-29.00)	7.1/5.3 (0.25-32.20)
Preoperative diagnosis				
Benign	174 (76.0%)	26 (78.8%)	11 (84.6%)	211 (76.7%)
Undetermined	10 (4.4%)	2 (6.1%)	0 (0%)	12 (4.4%)
Suspected malignant	40 (17.5%)	5 (15.2%)	1 (7.7%)	46 (16.7%)
Obvious malignant	5 (2.2%)	0 (0%)	1 (7.7%)	6 (2.2%)
Mass size (cm)	7.4 ± 4.5/6.4 (1.8 – 50.0)	6.7 ± 3.0/6.7 (2.0-18.40)	7.1 ± 3.2/7.3 (3.0-14.6)	7.3 ± 4.3/6.4 (1.8 – 50.0)
Carcinoma size (cm)	NA	0.44 (0.2-1.3) (10 cases)	0.6 (0.3-1.2) (3 cases)	NA
Elevated tumor makers	57 (24.9%)	9 (27.3%)	6 (46.2%)	72 (26.2%)
CA 125 (case/median)	47 / 62.0	7 / 169.0	5 / 89.9	59 / 71.2
CA125 (range, U/ml)	35.8 – 2263.5	45.7 – 1129.0	67.0-103.0	35.8 – 2263.5
CA19-9 (case/median)	20 / 97.0	2 / 241.4	NA	22 / 97.0
CA19-9 (range, U/ml)	41.4-480.0	91.4 – 391.3	NA	41.4-480.0
Ascites	39 (17.0%)	4 (12.1%)	1 (7.7%)	44 (16.0%)
Side of lesions (ovary)				
Left	95 (41.5%)	18 (54.5%)	8 (61.5%)	121 (44.0%)
Right	131 (57.2%)	15 (45.5%)	2 (15.4%)	146 (53.1%)
Bilateral	2 (0.9%)	0 (0%)	0 (0%)	2 (0.7%)
Metastatic	1 (0.4%)	0 (0%)	3 (23.1%)	6 (2.2%)

Abbreviations: SO, struma ovarii; OSC, ovarian strumal carcinoid; MSO, malignant struma ovarii; NA, not applicable

Notes: the age, follow-up time, and mass/carcinoid size were presented as mean ± standard deviation/median (range)

Combined imaging examinations, including ultrasound, CT and MRI, and serum tumor markers, were examined preoperatively to determine the nature of tumor. Most patients were considered as benign diseases, while only 6 cases manifested obvious malignant features. The median tumor size was 6.4 cm. Two patients (0.7%) had bilateral ovarian strumal diseases and 6 cases (2.2%) presented metastatic diseases at initial diagnosis. Ascites and elevated tumor markers were observed in 16.0% and 26.2% of cases, respectively. Some patients had more than one abnormal tumor marker. In this cohort, CA125 and CA19-9 were the two most frequently elevated tumor markers found in 59 (21.5%) and 22 (8.0%) patients, respectively. One-way ANOVA and χ^2^/Fisher’s exact test showed no statistical differences in terms of age, tumor sizes, the proportion of preoperatively suspected malignant, elevated tumor markers, and ascites.

**Fig. 1:**
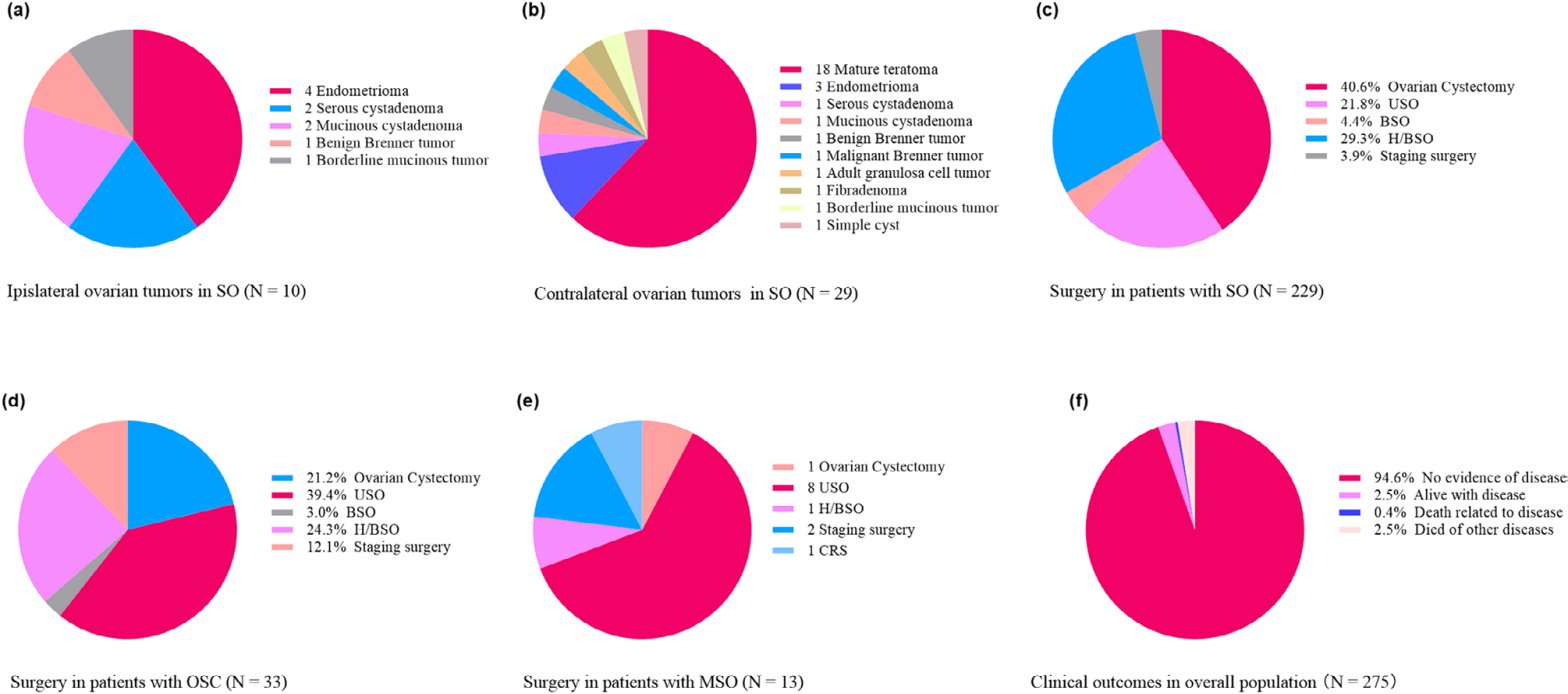
Clinical characteristics, surgical treatment, and clinical outcomes in this cohort. (**a**) coexisted ipsilateral ovarian tumors in SO; (**b**) contralateral ovarian tumors in SO; (**c**) surgery in OSC; (**d**) surgery in OSC; (**e**) surgery in MSO; (**f**) clinical outcomes

The details of the surgery and adjuvant therapy were described in subsequent subgroup analyses. During a median follow-up time of 5.3 years (range: 0.25–32.2), 9 patients had disease relapse. Of them, four patients underwent surgery, while the other five patients chose to follow up. At the final follow-up, 94.6% of the patients were alive with no evidence of disease (NED), 2.5% of them were alive with the disease (AWD), and 2.5% of them died of other diseases. Only one tumor-related death related to tumors occurred (DOD) (Fig. 1f).

### Subgroups analysis

#### Patients with SO

The median age was 43.0 years (range: 17–92), and predominantly affected patients were aged between 30 and 59 years (152 cases, 66.4%), but it was almost equally distributed in a 10-year interval. A large tumor size (≥ 10.0 cm) was detected in 39 (17.03%) patients, and a tumor size of over 15 cm was noted in 10 (4.37%) patients. Moreover, an 18-year-old girl had the largest tumor of 50 cm, originating from the right ovary. Only 5 patients were successfully preoperatively diagnosed with SO by imaging study, of which three patients were examined by ultrasound and the rest were by MRI (Table 1).

Although SO presented a right ovarian predominance (57.2% originated in right ovary), there were two patients had bilateral SO and one presented metastatic SO at their initial presentation. Elevated tumor markers were noted in approximately one-fourth of patients. Furthermore, the values of both CA125 and CA19-9 could be remarkably high that up to 2263.5 U/ml and 480.0 U/ml, respectively. Importantly, SO in 141 patients (61.6%) was pure without any other teratoma components. However, SO with adenomatous proliferation was noted in 15 cases (6.6%) in the overall cohort, including 3 cases of local adenomatous hyperplasia. Co-existence of other non-teratoma tumors in the ipsilateral ovary was rare and only found in 10 cases (4.4%), where endometrioma and cystadenoma were the most common pathology (Fig. 1a). However, SO with synchronous contralateral tumors was more frequent that found in 29 patients, of which mature teratoma (18 cases) was the most predominant (Fig. 1b). Besides, the mean Ki-67 index was nearly 2% in 26 patients who reported this item, with the highest value of only 8% and 92.3% (24/26) of patients had a Ki-67 index not more than 3%. However, no significant correlation could be found between a higher Ki-67 index and aggressive behavior of SO, such as metastasis.

Ovarian cystectomy was the most common treatment option administered in 40.6% of patients, followed by H/BSO (29.3%), USO (21.8%), and BSO (4.4%) (Fig. 1c; Table 1). Nonetheless, nine (3.9%) patients underwent comprehensive staging surgery, of which four were due to coexisting gynecologic cancer, and one due to a recurrent bilateral mucinous borderline ovarian tumor, and the rest four patients because of the uncertain intraoperative frozen pathology. Intraoperative frozen pathology was administered in 79 (34.5%) patients, of whom 61 (77.2%) was diagnosed with SO, and benign teratoma was identified in other 12 cases, but the rest 6 cases reported undetermined results or could not exclude malignancy. Two patients received postoperative adjuvant therapy due to coexisting rectal cancer.

Recurrence was suspected in 6 patients, but 4 patients presented ovarian cysts and chose to follow up without intervention. The other two patients developed metastatic SO at the peritoneum 11 years after the first surgery and at the anterior mesorectum 14 years after initial treatment, respectively. Surgical excision was then conducted, and they both achieved NED. After a median follow-up of 5.6 years (range: 0.4–32.2), all patients were alive, except seven patients who died of other diseases.

### Patients with OSC

In this cohort, OSC mostly affected women aged in the fourth decade (10 cases, 30.3%), followed by patients aged over 50 years (9 cases, 27.3%). Most OSC (78.8%) patients were preoperatively diagnosed with benign tumors. However, the mean carcinoid size (0.44 cm) was much smaller compared with the mass size (6.7 cm). All OSCs were unilateral diseases confined to the ovary and no carcinoid syndrome was noted (Table 1). Furthermore, 48.5% of the OSC contained only SO and carcinoid componence (pure OSC), and none coexisted with the ipsilateral or contralateral non-teratoma component. The Ki-67 index was reported in 18 patients, showing an extremely low value that the mean value was 2% and only three patients had a Ki-67 index of 5%.

USO was the most common surgical option administered in 39.4% of patients, followed by H/BSO (24.3%), ovarian cystectomy (21.2%), and comprehensive staging surgery (12.1%). Only 1 case (3.0%) underwent BSO (Fig. 1d). Notably, four patients received USO after a previous ovarian cystectomy that confirmed the diagnosis of OSC. Two patients were treated with platinum-based chemotherapy postoperatively, of whom one was due to coexisting ovarian adenocarcinoma.

The survival outcomes were excellent in the OSC patients. They all obtained NED and none experienced recurrences a median follow-up period of 3.1 years (range: 0.25–25.9).

### Patients with MSO (thyroid cancer arising in SO)

Imaging examinations had poor diagnostic efficiency in MSO patients, such that 11 (84.6%) patients were initially diagnosed with benign tumors, and the other two patients were suspected of epithelial ovarian carcinoma. Asymptomatic pelvic mass or ovarian cyst was the most common manifestation in these patients. The thyroid function test was within normal range.

The thyroid cancer components only occupied a small portion (0.6 cm) of the ovarian mass (mean size of 7.1 cm). At primary diagnosis, elevated thyroglobulin (TG) level was noted in one patient (7.7%) who had metastatic disease. However, three patients who had recurrent diseases showed remarkable higher TG levels, of whom two had TG > 1000 ng/ml. Conversely, the TG levels in these three patients were normal at primary diagnosis. Ten patients had MSO confined to the ovary at initial diagnosis, whereas the other three patients had metastatic diseases. Papillary thyroid carcinoma (PTC) was the most common pathologic subtype (6 cases, 46.2%), followed by follicular thyroid carcinoma (FTC) (5 cases, 38.5%). Follicular variants of PTC (FVPTC), mixed PTC and FTC were two less common subtypes that were identified in each patient. The mean Ki-67 index was about 5% in 7 reported cases, of which six patients exhibited no more than 5%, and one patient had the highest value of 15%. A genetic test was conducted inone patient with extensive metastatic disease and NRAS mutation was detected. Coexisted PTC in the neck was also found in one patient.

In patients with MSO, USO remained the most common surgical option administered in 61.5% (8 cases) of patients, including three patients who underwent USO after ovarian cystectomy. One patient who had multiple metastases in the peritoneum and left acetabulum underwent ovarian cystectomy, metastasectomy, and bone biopsy without adjuvant therapy to preserve fertility. One patient was treated with H/BSO. Staging/cytoreductive surgery was applied to three patients, of which one received USO followed by staging surgery (Fig. 1e). However, no intraoperative frozen pathology was applied in MSO patients and they all established the diagnosis by paraffin pathology. Lymphadenectomy was conducted in four patients, but all were negative. At initial treatment, adjuvant therapy was administered in 6 patients, including platinum-based chemotherapy in 5 patients and total thyroidectomy (TT) followed by radioiodine therapy (RAI) in another patient.

Eleven patients achieved NED after initial treatment and two were AWD. The patient who preserving fertility had successfully conceived and delivered at full term. In this patient, regular monitoring of serum TG showed a normal level, and imaging examinations of the thyroid and abdominopelvic cavity revealed stable disease. The patient with NRAS mutation metastasized to the liver and lung but maintained stable after receiving four courses of RAI. During the follow-up, three patients relapsed, with a 5-year and 10-year RFS rate of 88.9% and 71.1%, respectively, and the median RFS was 17.0 years. Repeat optimal cytoreductive surgery was administered in two patients who had multiple peritoneal metastases but showed a poor response to chemotherapy. One patient who developed multiple seedings in the peritoneum, liver, lung, and mediastinum eventually succumbed to the disease. The other patient received TT followed by RAI at 4 years after ceasing chemotherapy but showed only a partial response. The third patient who had disease recurrence in the lung 17 years after the first surgery, she achieved NED for more than 10 years after administered four courses of RAI.

After a median follow-up of 6.2 years (range: 0.8–29.0), the 10-year OS and DSS rates were both 75.0%, with only one death occurring 9.5 years after the diagnosis (Figure S2). Other nine patients achieved NED and three were AWD.

## Discussion

Our study demonstrated that 2.14% of ovarian teratoma could be classified as SO, of which 12.0% and 4.7% of SO may transform into OSC and MSO, repsectively. The overlapping imaging characteristics of benign and malignant tumors and their heterogeneous biological behaviors made the preoperative diagnosis of SO, OSC, and MSO quite challenging. Differential diagnosis among these diseases might be unpractical without pathology. The survival outcomes were excellent even after SO transformed into OSC or MSO.

The incidence of SO and its malignant transformations to OSC or MSO have been a puzzle for decades. Previous studies estimated that there were only about 300 cases of MSO, and researchers identified less than 150 cases of OSC, presuming that the rate of MSO arising in SO was nearly 5% [14–16]. Likewise, researchers estimated that approximately 5% of ovarian teratomas could be classified as SO [4, 10, 17]. Recently, Wei et al. identified 10 cases of MSO (10.4%) and 5 cases of OSC (5.2%) in a population of 96 SO patients, who first exhibited a crude malignant transformation rate in a large cohort [11]. Other cohort studies merely described the case numbers of SO, OSC, or MSO without indicating their incidence rates [12–14, 18]. We first described the incidence of SO in the background of ovarian teratoma and the rate of its transformation to OSC or MSO in the largest cohort to date. It was much lower than the previously presumed rate (2% vs. 5%) but could be explained by the paucity of cohort studies of SO. Furthermore, the OSC arising in SO was more frequent (12.0%) than a previous study (5.2%) [11], but the result conversed in the MSO (4.7% vs. 10.4%). The inconsistent results may be attributed to the differences in sample sizes and the selection bias since some OSC and MSO patients were referred to our hospital for further treatments after their initial diagnoses, which could have overestimated the exact incidence rate.

We found that only five patients were preoperative correctly diagnosed as SO but none correctly diagnosed before surgery in OSC and MSO, indicating the extreme challenges in the precision diagnosis of this disease. Unlike common ovarian teratomas, neither ultrasonography nor CT, or MRI showed satisfactory efficacy in the identification of SO with or without serum tumor markers [4, 19–21]. Besides, some patients with SO presented elevated CA125 levels and/or remarkable ascites that highly suspected epithelial ovarian carcinoma (EOC) [3, 21]. Our study showed that one-fifth of the patients were suspected of malignancy either in the SO subgroup or the overall population, and even metastasis at initial presentation could be observed in the SO subgroup. This dilemma was much more obvious that OSC and MSO were usually small, and focal malignant transformations underlying the predominant SO components. Routine use of intraoperative frozen pathology in suspected ovarian stumal diseases or undetermined ovarian mass may be a practical option to optimize the diagnosis and treatment. We found that frozen pathological examination could correctly diagnose 77.2% of SO cases in 79 patients and only 7.6% of patients reported undefined results, of whom four were SO with adenomatous proliferation identified in paraffin pathology. Due to the satisfactory outcomes, even for OSC and MSO, a proper surgical strategy can still be conducted based on this evidence.

The treatment of SO can certainly be referred to the common strategy conducted in mature ovarian teratomas. However, as SO patients usually present larger solid-cystic masses which can potentially transform to OSC and MSO, whether ovarian cystectomy or USO could be a preferred option should be thoroughly considered based on the coexisting gynecologic diseases, fertility desire, and the residual function of the involved ovary. Similarly, USO may be a better surgical option in patients with OSC confined to the ovary, while ovarian cystectomy is also practical in whom previously underwent contralateral oophorectomy and have fertility desire and have previously received [6, 12, 14]. However, treatment for patients with metastatic OSC remains controversial. Adjuvant chemotherapy may be reserved for recurrent or metastatic OSC that could not be completely resected [22]. Nevertheless, OSC may be insensitive to chemotherapy due to low proliferative nature, and radiotherapy may be more suitable to achieve disease remission [14]. Therefore, USO and metastasectomy with personalized adjuvant therapies might be considered in such cases [14, 23].

The treatment of MSO has always been controversial. Conservative surgery alone or aggressive treatments, including comprehensive staging/cytoreductive surgery with combined TT and postoperative RAI, have been proposed in previous studies [9, 24–26]. However, unlike EOC, the survival outcomes of patients with MSO were excellent and the 10-year DSS rate was approximately 90% in MSO, and patients with MSO confined to the ovary had better outcomes [9, 18]. Previous studies have evaluated the impact of different surgical options on survival outcomes and the necessity of postoperative adjuvant therapy in patients with metastatic MSO, MSO confined to the ovary [25]. Although it remains undetermined, most studies indicated the importance of balancing the radicality of surgery and long-term quality of life [9, 25]. Our current results supported these findings that the survival outcomes were satisfactory after long-term follow-up, irrespective of the varied treatment strategies employed.

Previous studies have shown that the recurrence rate in MSO confined to the ovary was low and the efficacy of RAI in improving the survival outcomes in these patients was uncertain [9, 27]. Nevertheless, RAI was widely recommended in metastatic MSO for ablation of residual diseases [25, 26, 28], and it may be the only practical therapeutic option in patients with unresectable diseases, such as multiple liver, lung, or bone metastases [29]. Although the exact therapeutic response rate for RAI in MSO was unavailable due to the extreme rarity and most patients received both surgery with RAI [15]. Some patients achieved stable diseases or complete remission with RAI therapy alone [30–33]. However, TT was mandatory before RAI which led to lifetime thyroxine replacement therapy and RAI could potentially impair ovarian function in childbearing patients [34]. Therefore, RAI should be routinely performed in metastatic MSO and individually applied in MSO confined to the ovary. However, due to the histological nature of MSO, chemotherapy has been shown poor response in this population, while radiotherapy might be an option for RAI-refractroy MSO referring to thyroid cancer [29, 35].

The large sample size in this study strengthened the reliability and feasibility to improve the clinical practice in managing patients with ovarian strumal diseases. The retrospective nature of this study and the relative rarity of OSC and MSO were the two main limitations. Future studies should emphasize on exploring validated preoperative diagnostic methods in this population.

## Conclusion

2.14% of ovarian teratoma could be classified as SO, of which 12.0% and 4.7% of SO may transform into OSC and MSO, repsectively. The clinical features slightly varied among SO, OSC, and MSO, and preoperative identification or differential diagnosis based on their clinical or imaging characteristics could be challenging. The survival outcomes were excellent even after SO malignant transformation to OSC or MSO.

### Electronic supplementary material

Below is the link to the electronic supplementary material.

**Supplementary Figure S1**. The inclusion process of patients with ovarian strumal diseases in this study.

**Supplementary Figure S2.** DSS and RFS in patients with MSO.

**Supplementary Table S1**. The detailed surgical options in patients with SO, OSC, and MSO.

## Data Availability

All data generated or analyzed during this study are included in this published article and supplementary files. The datasets used and/or analyzed during the current study can be obtained from the corresponding author upon reasonable request.

## Abbreviations

SO: struma ovarii
OSC: ovarian strumal carcinoid
MSO: malignant struma ovarii
USO: unilateral salpingo-oophorectomy
BSO: bilateral salpingo-oophorectomy
H/BSO: hysterectomy with bilateral salpingo-oophorectomy
TG: thyroglobulin
TT: total thyroidectomy
RAI: radioidine therapy

## References

[1] Outwater EK, Siegelman ES, Hunt JL (2001). Ovarian teratomas: tumor types and imaging characteristics. Radiographics.

[2] Devaney K, Snyder R, Norris HJ, Tavassoli FA (1993). Proliferative and histologically malignant struma ovarii: a clinicopathologic study of 54 cases. Int J Gynecol Pathol.

[3] Wang S, He X, Yang H, Chen L (2022). Struma Ovarii Associated with Ascites and elevated CA125: two case reports and review of the literature. Int J Womens Health.

[4] Savelli L, Testa AC, Timmerman D (2008). Imaging of gynecological disease (4): clinical and ultrasound characteristics of struma ovarii. Ultrasound Obstet Gynecol.

[5] Mui MP, Tam KF, Tam FK, Ngan HY (2009). Coexistence of struma ovarii with marked ascites and elevated CA-125 levels: case report and literature review. Arch Gynecol Obstet.

[6] Turla A, Zamparini M, Milione M (2022). Ovarian strumal carcinoid: Case Report, systematic literature review and pooled analysis. Front Endocrinol (Lausanne).

[7] Zhang X, Axiotis C (2010). Thyroid-type carcinoma of struma ovarii. Arch Pathol Lab Med.

[8] Muller KE, Tafe LJ, Gonzalez JL, West LA, Schned AR (2015). Ovarian strumal carcinoid producing peptide YY associated with severe constipation: a case report and review of the literature. Int J Gynecol Pathol.

[9] Marti JL, Clark VE, Harper H (2012). Optimal surgical management of well-differentiated thyroid cancer arising in struma ovarii: a series of 4 patients and a review of 53 reported cases. Thyroid.

[10] Ikeuchi T, Koyama T, Tamai K (2012). CT and MR features of struma ovarii. Abdom Imaging.

[11] Wei S, Baloch ZW, LiVolsi VA (2015). Pathology of Struma Ovarii: a report of 96 cases. Endocr Pathol.

[12] Robboy SJ, Scully RE (1980). Strumal carcinoid of the ovary: an analysis of 50 cases of a distinctive tumor composed of thyroid tissue and carcinoid. Cancer.

[13] Theurer S, Ingenwerth M, Herold T, Herrmann K, Schmid KW (2020). Immunohistochemical Profile and 47-Gene next-generation sequencing (NGS) solid Tumor Panel Analysis of a series of 13 Strumal Carcinoids. Endocr Pathol.

[14] Li S, Wang X, Sui X (2022). Clinical characteristics and survival outcomes in patients with ovarian strumal carcinoid. BMC Cancer.

[15] Li S, Kong S, Wang X (2021). Survival outcomes and prognostic predictors in patients with malignant struma Ovarii. Front Med (Lausanne).

[16] Siegel MR, Wolsky RJ, Alvarez EA, Mengesha BM (2019). Struma ovarii with atypical features and synchronous primary thyroid cancer: a case report and review of the literature. Arch Gynecol Obstet.

[17] Roth LM, Talerman A (2007). The enigma of struma ovarii. Pathology.

[18] Goffredo P, Sawka AM, Pura J (2015). Malignant struma ovarii: a population-level analysis of a large series of 68 patients. Thyroid.

[19] Dujardin MI, Sekhri P, Turnbull LW (2014). Struma ovarii: role of imaging?. Insights Imaging.

[20] Shen J, Xia X, Lin Y, Zhu W, Yuan J (2011). Diagnosis of Struma ovarii with medical imaging. Abdom Imaging.

[21] Fujiwara S, Tsuyoshi H, Nishimura T, Takahashi N, Yoshida Y (2018). Precise preoperative diagnosis of struma ovarii with pseudo-meigs’ syndrome mimicking ovarian cancer with the combination of (131)I scintigraphy and (18)F-FDG PET: case report and review of the literature. J Ovarian Res.

[22] Kong S, Sun J, Sui X et al. Clinical characteristics and survival outcomes in patients with primary ovarian carcinoid: a historical cohort study. Acta Obstet Gynecol Scand. 2023. 10.1111/aogs.14578 PMC10333664 37059424

[23] Kurabayashi T, Minamikawa T, Nishijima S (2010). Primary strumal carcinoid tumor of the ovary with multiple bone and breast metastases. J Obstet Gynaecol Res.

[24] Jean S, Tanyi JL, Montone K (2012). Papillary thyroid cancer arising in struma ovarii. J Obstet Gynaecol.

[25] DeSimone CP, Lele SM, Modesitt SC (2003). Malignant struma ovarii: a case report and analysis of cases reported in the literature with focus on survival and I131 therapy. Gynecol Oncol.

[26] Shrimali RK, Shaikh G, Reed NS (2012). Malignant struma ovarii: the west of Scotland experience and review of literature with focus on postoperative management. J Med Imaging Radiat Oncol.

[27] Li S, Yang T, Xiang Y (2021). Clinical characteristics and survival outcomes of malignant struma ovarii confined to the ovary. BMC Cancer.

[28] McGill JF, Sturgeon C, Angelos P (2009). Metastatic struma ovarii treated with total thyroidectomy and radioiodine ablation. Endocr Pract.

[29] Li S, Yang T, Li X (2020). FIGO Stage IV and Age over 55 years as prognostic predicators in patients with metastatic malignant struma Ovarii. Front Oncol.

[30] Zekri JM, Manifold IH, Wadsley JC (2006). Metastatic struma ovarii: late presentation, unusual features and multiple radioactive iodine treatments. Clin Oncol (R Coll Radiol).

[31] Janszen EW, van Doorn HC, Ewing PC (2008). Malignant struma ovarii: good response after thyroidectomy and I ablation therapy. Clin Med Oncol.

[32] Cong H, Li T, Chen G (2015). Missed initial diagnosis of Malignant Struma Ovarii containing follicular thyroid carcinoma: metastatic pulmonary recurrence 17 year after Ovariectomy. Int J Gynecol Pathol.

[33] Tzelepis EG, Barengolts E, Garzon S, Shulan J, Eisenberg Y (2019). Unusual case of malignant struma Ovarii and cervical thyroid Cancer preceded by ovarian teratoma: Case Report and Review of the literature. Case Rep Endocrinol.

[34] van Velsen EFS, Visser WE, van den Berg SAA (2020). Longitudinal analysis of the Effect of Radioiodine Therapy on Ovarian Reserve in females with differentiated thyroid Cancer. Thyroid.

[35] Ukita M, Nakai H, Kotani Y (2014). Long-term survival in metastatic malignant struma ovarii treated with oral chemotherapy: a case report. Oncol Lett.

