# Supplementary figures and images for: Incidence, clinical characteristics, and survival outcomes of ovarian strumal diseases: a retrospective cohort study
Source: BMC Womens Health. 2023 Sep 19;23:497. doi: 10.1186/s12905-023-02624-5 (PMC10510205; doi:10.1186/s12905-023-02624-5)

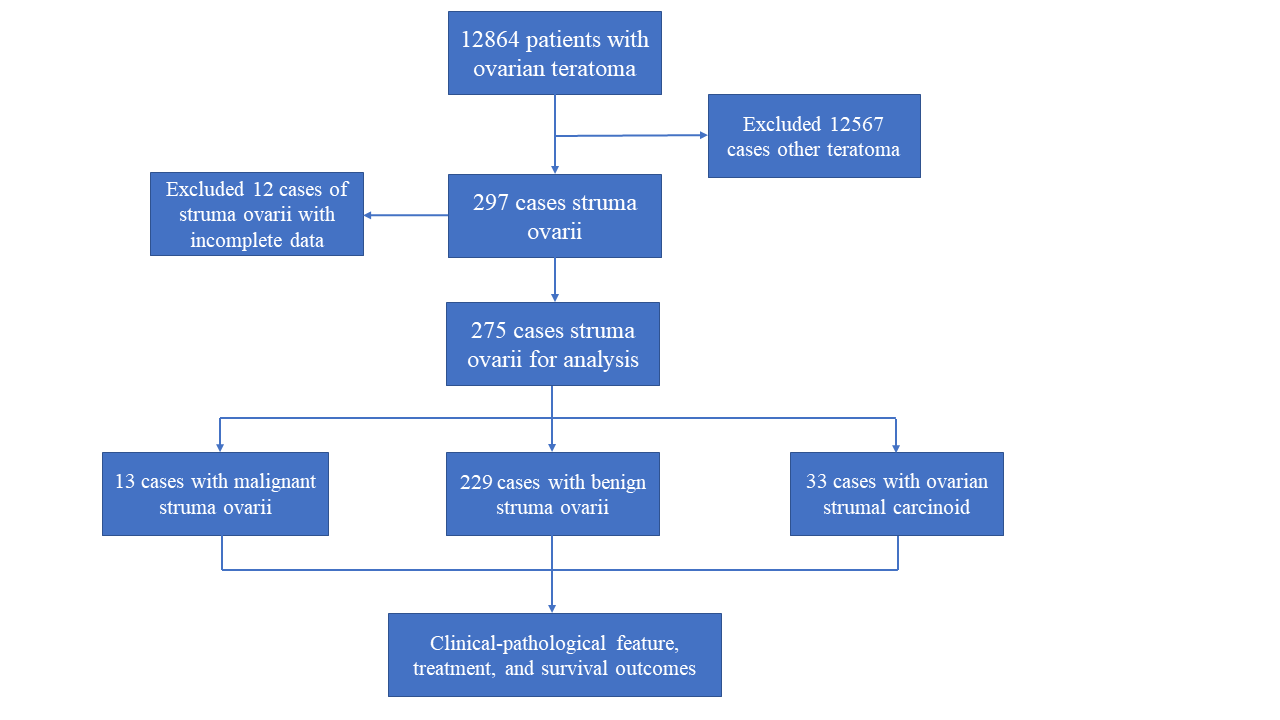

Supplement: Supplementary file 1 — Supplementary Figure S1. The inclusion process of patients with ovarian strumal diseases in this study. [file 12905_2023_2624_MOESM1_ESM.tif]

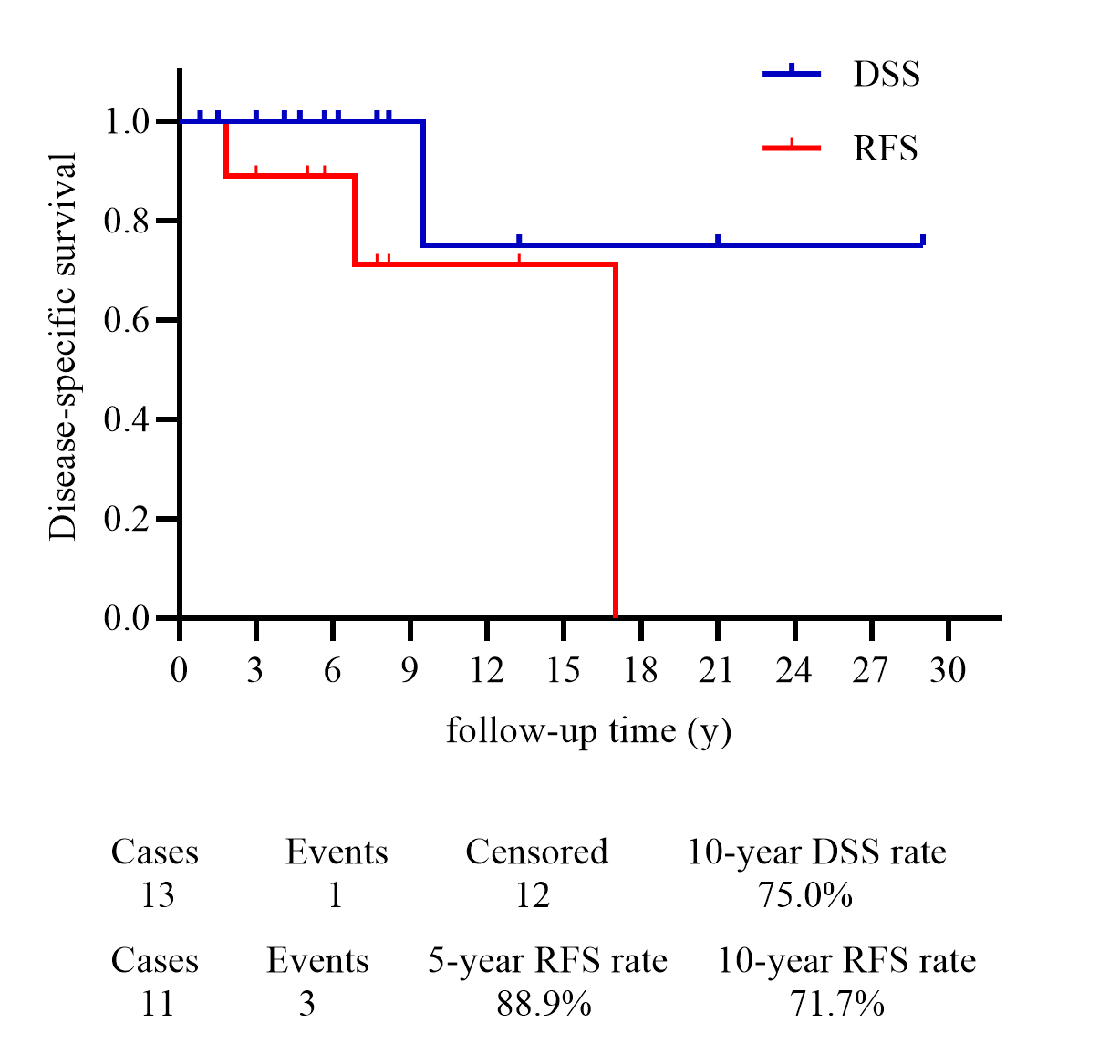

Supplement: Supplementary file 2 — Supplementary Figure S2. DSS and RFS in patients with MSO. [file 12905_2023_2624_MOESM2_ESM.tif]
