# Supplementary material for: Incidence, clinical characteristics, and survival outcomes of ovarian strumal diseases: a retrospective cohort study
Source: BMC Womens Health. 2023 Sep 19;23:497. doi: 10.1186/s12905-023-02624-5 (PMC10510205; doi:10.1186/s12905-023-02624-5)
Supplement: Supplementary file 3 — Supplementary Table S1. The detailed surgical options in patients with SO, OSC, and MSO. [file 12905_2023_2624_MOESM3_ESM.docx]

|  | SO  (N = 229, 83.3%) | OSC  (N = 33, 12.0%) | MSO  (N = 13, 4.7%) |
| --- | --- | --- | --- |
| Ovarian cystectomy | 93 (40.6%) | 7 (21.2%) | 1 (7.7%) |
| metastasectomy | 0 | 0 | 1 |
| USO | 50 (21.8%) | 13 (39.4%) | 8 (61.5%) |
| Appendectomy | 1 (2.0%) | 0 | 1 (12.5%) |
| Omentectomy | 3 (6.0%) | 0 | 2 (25.0%) |
| Lymphadenectomy | 1 (2.0%) | 0 | 1 (12.5%) |
| metastasectomy | 1 (2.0%) | 0 | 0 |
| BSO | 10 (4.4%) | 1 (3.0%) | 0 (0%) |
| Appendectomy | 1 | 0 | 0 |
| Omentectomy | 1 | 0 | 0 |
| H/BSO | 67 (29.3%) | 8 (24.2%) | 1 (7.7%) |
| Appendectomy | 1 | 0 | 0 |
| Staging/CRS | 9 (3.9%) | 4 (12.1%) | 3 (23.1%) |
| Index surgery | 1 (0.4%) | 4 (12.1%) | 5 (38.5%) |
| Cystectomy | 1 | 4 | 4 |
| USO | 0 | 0 | 1 |

Table S1. The detailed of surgical options in patients with SO, OSC, and MSO.

Abbreviations: SO, struma ovarii; OSC, ovarian strumal carcinoid; MSO, malignant struma ovarii; USO, unilateral salpingo-oophorectomy; BSO, bilateral salpingo-oophorectomy; H/BSO, hysterectomy with bilateral salpingo-oophorectomy; CRS, cytoreductive surgery.
